# Correlation of MDR1 gene polymorphism with propofol combined with remifentanil anesthesia in pediatric tonsillectomy

**DOI:** 10.18632/oncotarget.23168

**Published:** 2017-12-12

**Authors:** YunLong Zhang, Yongpei Li, Hongfa Wang, Fang Cai, Sheliang Shen, Xiaopan Luo

**Affiliations:** ^1^ Department of Anesthesiology, Zhejiang Provincial People’s Hospital, People’s Hospital of Hangzhou Medical College, Hangzhou, Zhejiang, China; ^2^ Hangzhou Women’s Hospital, Hangzhou Maternity and Child Health Care Hospital, Hangzhou, Zhejiang, China

**Keywords:** multidrug resistance gene, single nucleotide polymorphism, propofol, remifentanil, tonsillectomy

## Abstract

The motive of this study was to investigate the interaction between polymorphisms in the MDR1 gene and anesthetic effects following pediatric tonsillectomy. In total, 240 children undergoing tonsillectomy with preoperative propofol-remifentanil anesthesia were selected. Genomic DNA was extracted from the peripheral blood of children after operation, and the MDR1 gene polymorphisms of 2677 G>T/A, 1236 C>T and 3435 C>T were detected by direct sequencing. We tested mean arterial pressure, diastolic blood pressure, systolic blood pressure, and heart rate at several time-points: T0 (5 mins after the repose), T1 (0 min after tracheal intubation), T2 (5 mins after the tracheal intubation), T3 (0 min after the tonsillectomy), T4 (0 min after removal of the mouth-gag) and T5 (5 min after the extubation). The visual analog scale, the face, legs, activity, cry, and consolability pain assessment, and the Ramsay sedation score were recorded after the patients regained consciousness. Adverse reactions were also recorded. The time of induction, respiration recovery, eye-opening, and extubation of children with the CC genotype were found to be shorter compared to the CT + TT genotype of MDR1 1236C > T (all P <.05). The mean arterial pressure, diastolic blood pressure, systolic blood pressure, and heart rate were significantly reduced at T5 in children with the CC genotype (all P <.05). The visual analog scale at 1, 2, 4, and 8 hours post-operation, and the Ramsay sedation score at 5, 10, and 30 min after the extubation were decreased, while the face, legs, activity, cry, and consolability pain assessment score increased (all P <0.05). There was no statistically significant difference in the adverse reaction of MDR1 mutations (P> 0.05). It could be concluded that anesthetic effect following pediatric tonsillectomy in patients with the MDR1 1236C > T CC genotype was stronger than in those carrying the CT + TT genotype.

## INTRODUCTION

Tonsillectomy is known to be one of the most common and severely painful surgeries that children undergo1 [1]. It is conducted inside the oral cavity, and strict anesthesia requirements are needed to eliminate undesirable reflection phenomenon occurring during surgery since it easily leads to throat irritation [2]. In addition, it is conducive to post-operative recovery to maintain stable vital signs in children during surgery since the operation time is short [3]. The tonsils are rich in blood and large amounts of secretions, so more attention should be paid to anesthesia to avoid accidental inhalation during surgery [4]. With recent advancements in anesthetic and surgical techniques, tonsillectomy has become safer than in older times. The 2 main narcotic drugs are propofol and remifentanil [5]. Both are ultra-short-acting opioids that have less impact on post-operative recovery and are considered safe [6]. However, studies have shown that nausea, vomiting, muscle stiffness and dose-dependent heart rate (HR) increase and systolic blood pressure decreases after anesthesia, and increased post-operative analgesia is needed because of acute opioid tolerance [7]. MDR1 encodes a 170-kDa transmembrane protein, P-glycoprotein (P-gp). P-gp is an efflux pump for a variety of lipophilic compounds. [8, 9]. MDR1 is expressed in many tissues [10], and many single-nucleotide polymorphisms (SNPs) have been identified in the exons of MDR1. Among these SNPs, 1236C > T, 2677G > T/A, and 3435C > T in MDR1 are the main variants that have been investigated [11]. Our study aims to investigate the role of the MDR1 gene polymorphisms 1236C > T, 2677G > T/A, and 3435C > T in the anesthetic effects of propofol combined with remifentanil. We hope to provide knowledge for better anesthetic effects in pediatric tonsillectomy.

## RESULTS

### Allele frequencies of MDR1

A total of 240 subjects were recruited for this study. According to the sequencing results, the number of wild-type heterozygote patients for MDR1 2677G>T/A with TG, GA, and TA was 101, 31, and 30 respectively, and the number of mutant homozygote patients with TT, GG and AA was 30, 42, and 6 respectively. The number of patients with MDR1 1236C > T wild homozygote CC, heterozygote CT, and the mutational homozygote TT was 25, 118, and 91, respectively. The number of patients with MDR1 3435 C > T wild homozygote CC, heterozygote CT, and mutant homozygote TT was 92, 18, and 40, respectively.

**Figure 1:**
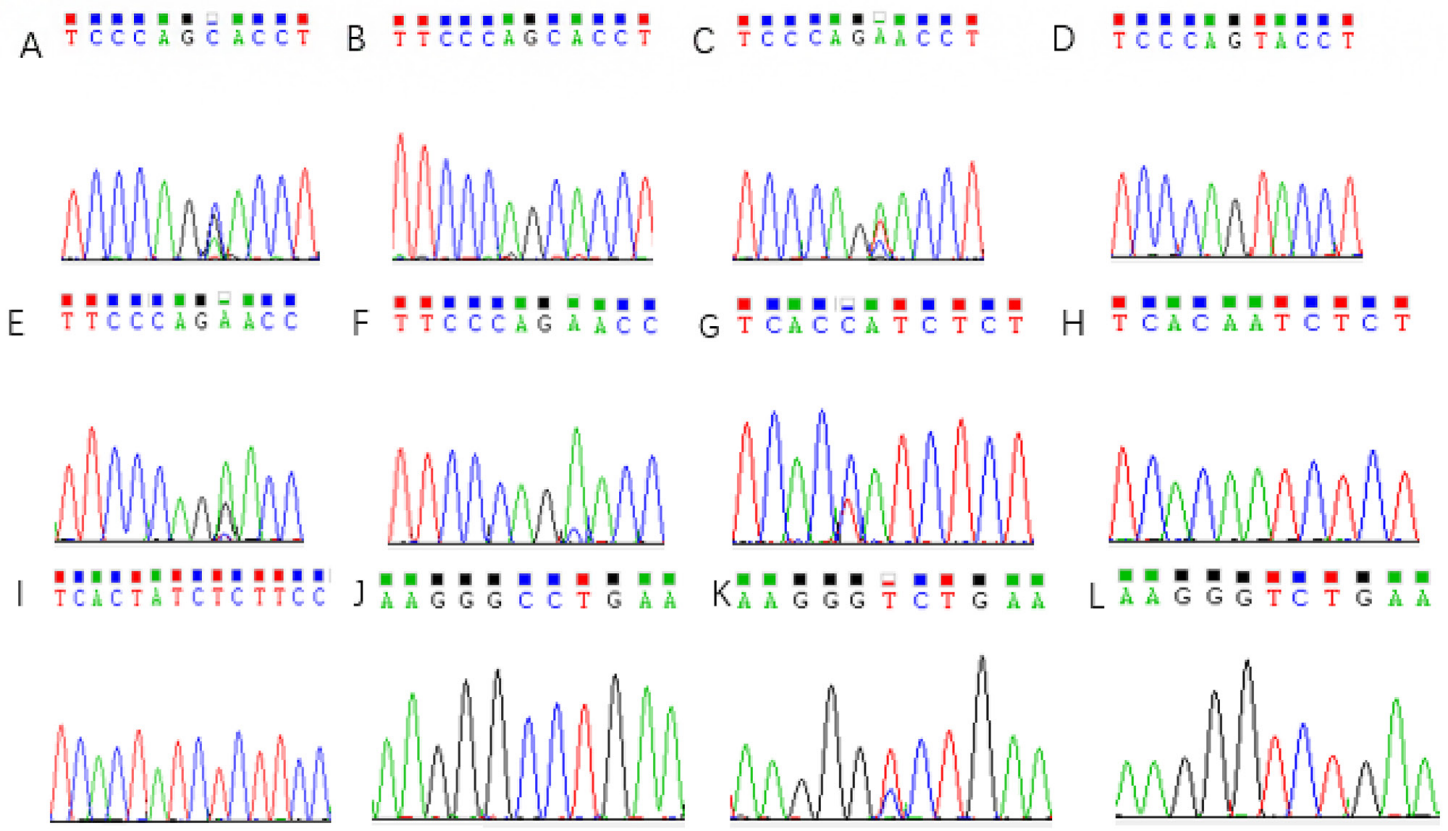
Sequencing results of 2677 G>T/A mutations site in MDR1 gene **(A)** 2677 G>T/A GA genotype; **(B)** 2677 G>T/A GG genotype; **(C)** 2677 G>T/A TA genotype; **(D)** 2677 G>T/A AA genotype; **(E)** 2677 G>T/A TG genotype; **(F)** 2677 G>T/A TT genotype; **(G)** 3435C>T CT genotype; **(H)** 3435C>T CC genotype; **(I)** 3435C>T TT genotype; **(J)** 1236C>T CC genotype; **(K)** 1236C>T CT genotype; **(L)** 1236C>T TT genotype.

The MDR1 gene distribution for 3435C > T, 1236C > T, and 2677G > T/A was consistent with Hardy–Weinberg equilibrium (all P >0.05), which indicated that the gene distribution of MDR1 3435C > T, 1236C > T, and 2677G > T/A achieved genetic equilibrium. It was then recorded and represented in Table 1. The sequencing results for each mutant site are shown in Figure 1.

**Table 1:** Comparison of gene frequencies between each loci of MDR1 gene

Gene	Theoretical frequency (%)	Actual frequency (%)	*χ*^*2*^	*P*
2677 G>T/A			0.82	0.98
TT	30 (12.50)	28 (11.67)		
TG	101 (42.08)	108 (45.00)		
GG	42 (17.50)	39 (16.25)		
GA	31 (12.92)	34 (14.17)		
TA	30 (12.50)	27 (11.25)		
AA	6 (2.50)	4 (1.67)		
1236C>T			0.89	0.64
CC	25 (10.42)	30 (12.50)		
CT	118 (49.17)	109 (45.42)		
TT	97 (40.42)	101 (42.08)		
3435C>T			0.30	0.86
CC	92 (38.33)	88 (36.67)		
CT	108 (45.00)	114 (47.50)		
TT	40 (16.67)	38 (15.83)		

### Effect of MDR1 gene polymorphisms on anesthesia induction and restoration

No significant differences were found between different genotypes on MDR1 2677G > T/A concerning the time of induction, respiration recovery, eye-opening, and extubation (all *P* >0.05). No significant difference was revealed between MDR1 3435C > T CC and CT + TT genotypes that increased the time of induction, respiration recovery, eye-opening, and extubation (all *P* >0.05). Compared to the MDR1 1236C > T CC genotype, children bearing the CT + TT genotype exhibited longer times of induction, respiration recovery, eye-opening, and extubation (all *P* <0.05), as demonstrated in Table 2.

**Table 2:** Comparison of anesthesia induction and restoration time between different MDR1 genotypes patients$\left(\bar{x}\pm s\right)$

Genotype	N	Time of induction (min)	Time of respiration recovery (min)	Time of eye-opening (min)	Time of extubation (min)
2677 G>T/A					
TT	28	3.84±0.32	5.87±0.61	8.87±0.62	8.67±0.65
TG	108	3.89±0.46	5.92±0.62	8.91±0.72	8.74±0.68
GG	39	3.91±0.54	5.80±0.71	8.84±0.65	8.77±0.74
GA	34	3.90±0.61	5.90±0.59	8.90±0.67	8.73±0.67
TA	27	4.01±0.58	2.78±0.54	9.01±0.79	8.75±0.81
AA	4	3.93±0.57	5.74±0.63	8.84±0.81	8.79±0.78
1236C>T					
CC	30	3.75±0.45	5.42±0.46	8.47±0.68	8.37±0.71
CT+ TT	210	4.12±0.37^▲^	5.86±0.48^▲^	8.92±0.72^▲^	8.76±0.75^▲^
3435C>T					
CC	88	3.98±0.48	5.96±0.74	8.83±0.81	8.78±0.71
CT+ TT	152	3.94±0.44	5.93±0.68	8.86±0.84	8.72±0.81

N=number

^▲^ *P*<0.05 compared with the CC genotype.

### Effect of MDR1 gene polymorphisms on hemodynamics after extubation

The MAP, SBP, DBP, and HR of patients with different genotypes at T1, T2, T3, T4, and T5 were found to be lower than those at T0 (all *P* <0.05). There was no significant difference revealed between the MDR1 3435C > T CC and CT + TT genotypes i MAP, SBP, DBP, and HR at T0, T1, T2, T3, T4, and T5 (all *P* >0.05). This was also applicable for the different genotypes on MDR1 2677G > T/A (all *P* >0.05). Compared to the MDR1 1236C > T CT + TT genotype, the children with the CC genotype exhibited significantly reduced MAP, SBP, DBP, and HR at T5 (all *P* <0.05) and nonsignificant differences during other time points (all *P* >0.05), as demonstrated in Table 3.

**Table 3:** Comparison of hemodynamics parameters of different mdr1 genotype patients$\left(\bar{x}\pm s\right)$

Indexes	Genotype	N	*T*_*0*_ (min)	*T*_*1*_ (min)	*T*_*2*_ (min)	*T*_*3*_ (min)	*T*_*4*_ (min)	*T*_*5*_ (min)
	2677 G>T/A							
	TT	28	86.87±7.46	80.65±5.86^●^	80.35±6.52^●^	76.32±6.58^●^	75.61±8.26^●^	69.63±6.13^●^
	TG	108	85.38±7.54	80.36±7.65^●^	81.36±8.41^●^	75.74±7.31^●^	75.21±6.31^●^	70.31±6.61^●^
	GG	39	86.55±7.45	80.63±7.18^●^	78.42±6.32^●^	76.76±7.36^●^	75.89±7.46^●^	72.35±7.50^●^
	GA	34	87.12±7.86	78.46±7.13^●^	78.36±8.67^●^	76.74±6.63^●^	76.12±7.64^●^	70.71±6.36^●^
MAP	TA	27	88.05±7.91	80.23±4.48^●^	79.85±8.26^●^	78.53±7.36^●^	71.41±6.95^●^	70.46±6.31^●^
	AA	4	90.52±8.15	77.82±6.31^●^	77.32±6.45^●^	76.81±5.73^●^	75.51±5.61^●^	70.34±5.76^●^
	1236C>T							
	CC	30	85.51±7.04	78.71±6.54^●^	78.71±6.24^●^	78.71±7.24^●^	73.58±8.15^●^	67.49±5.42^●^
	CT+ TT	210	86.04±8.74	84.05±8.75^●^	82.81±8.55^●^	77.54±7.42^●^	74.12±7.55^●^	73.45±7.79^●★^
	3435C>T							
	CC	88	85.27±8.75	80.44±7.88^●^	79.78±6.54^●^	77.01±7.24^●^	71.77±7.05^●^	69.04±5.47^●^
	CT+ TT	152	84.27±7.49	80.01±8.01^●^	79.48±7.31^●^	76.42±6.89^●^	73.05±6.54^●^	70.52±6.15^●^
	2677 G>T/A							
	TT	28	168.12±15.85	156.41±20.54^●^	155.74±14.75^●^	148.24±13.85^●^	141.68±12.74^●^	138.24±14.02^●^
SBP	TG	108	167.02±15.74	160.12±17.52^●^	152.78±15.98^●^	147.91±1502^●^	146.87±15.01^●^	137.02±12.54^●^
	GG	39	168.65±18.96	157.43±15.24^●^	156.42±20.15^●^	150.24±17.05^●^	148.97±14.02^●^	138.55±14.02^●^
	GA	34	168.79±17.14	155.97±16.75^●^	154.85±9.14^●^	145.04±20.14^●^	149.02±14.52^●^	137.01±17.05^●^
	TA	27	165.24±18.24	152.54±14.25^●^	150.41±1.65^●^	151.04±16.52^●^	143.75±14.02^●^	136.85±12.58^●^
	AA	4	175.98±18.95	149.01±12.52^●^	147.58±9.88^●^	147.22±15.87^●^	144.62±11.02^●^	138.41±11.14^●^
	1236C>T							
	CC	30	170.55±18.78	156.47±13.52^●^	154.38±12.25^●^	147.24±15.36^●^	144.01±17.31^●^	132.64±10.25^●^
	CT+ TT	210	167.14±16.02	159.85±18.04^●^	153.65±15.77^●^	148.42±15.87^●^	141.75±13.25^●^	143.05±13.52^●★^
	3435C>T							
	CC	88	164.87±17.85	157.65±13.05^●^	153.48±12.87^●^	146.51±15.02^●^	140.02±13.85^●^	141.35±12.58^●^
	CT+ TT	152	167.84±15.03	160.25±14.77^●^	152.98±14.97^●^	150.01±14.01^●^	141.55±13.05^●^	140.21±13.24^●^
	2677 G>T/A							
	TT	28	99.01±8.59	92.25±7.89^●^	92.15±8.77^●^	91.68±9.14^●^	91.25±8.77^●^	90.04±8.79^●^
	TG	108	97.12±10.54	93.54±9.02^●^	93.54±9.14^●^	92.79±8.54^●^	89.14±8.75^●^	88.77±9.01^●^
	GG	39	98.37±8.55	92.65±7.87^●^	91.55±8.75^●^	90.64±9.88^●^	89.25±9.32^●^	88.94±8.34^●^
	GA	34	100.05±8.74	93.54±7.83^●^	92.25±4.76^●^	92.32±4.85^●^	91.15±7.71^●^	86.54±8.97^●^
DBP	TA	27	99.38±7.98	9.25±8.79^●^	90.89±8.05^●^	89.65±10.25^●^	88.76±8.65^●^	87.19±7.69^●^
	AA	4	108.12±7.94	92.43±6.54^●^	91.84±5.78^●^	90.48±6.78^●^	87.91±7.45^●^	85.78±7.15^●^
	1236C>T							
	CC	30	98.97±6.39	92.01±6.85^●^	91.58±9.14^●^	91.58±8.77^●^	87.65±8.84^●^	80.14±3.58^●^
	CT+ TT	210	97.21±8.55	92.78±9.42^●^	94.05±9.87^●^	93.45±8.81^●^	87.67±8.99^●^	87.24±7.54^●★^
	3435C>T							
	CC	88	98.37±8.75	94.15±10.25^●^	93.77±8.68^●^	93.25±7.76^●^	90.28±7.58^●^	88.7±9.24^●^
	CT+ TT	152	96.24±8.05	92.97±8.79^●^	92.75±9.43^●^	92.35±9.71^●^	90.14±8.75^●^	86.65±8.21^●^
	2677 G>T/A							
HR	TT	28	94.73±9.21	81.24±9.75^●^	82.05±7.05^●^	76.02±7.64^●^	73.54±7.15^●^	75.78±6.02^●^
	TG	108	96.54±9.41	79.65±8.01^●^	79.68±7.54^●^	78.54±6.75^●^	73.24±6.85^●^	78.35±6.24^●^
	GG	39	96.8±10.24	79.62±7.58^●^	77.68±8.79^●^	76.65±7.43^●^	72.43±7.15^●^	76.02±7.21^●^
	GA	34	96.89±10.24	81.58±7.45^●^	82.54±6.78^●^	78.05±6.84^●^	73.85±7.25^●^	77.89±7.24^●^
	TA	27	97.78±9.41	82.03±7.54^●^	80.94±7.78^●^	77.79±8.02^●^	72.68±7.15^●^	77.79±7.21^●^
	AA	4	97.85±6.68	83.56±8.14^●^	75.01±10.25^●^	75.43±8.01^●^	69.64±6.38^●^	75.81±8.01^●^
	1236C>T							
	CC	30	95.02±8.02	85.24±7.31^●^	84.68±9.78^●^	82.03±8.78^●^	74.25±7.25^●^	67.54±7.02^●^
	CT+ TT	210	95.89±9.77	86.45±8.24^●^	83.65±8.41^●^	82.05±7.85^●^	75.1±7.05^●^	74.51±7.51^●★^
	3435C>T							
	CC	88	96.54±9.78	81.64±7.58^●^	79.65±8.15^●^	76.24±7.54^●^	73.02±8.54^●^	71.25±602^●^
	CT+ TT	152	96.38±8.91	86.65±8.79^●^	78.14±7.98^●^	75.55±6.85^●^	73.15±6.48^●^	72.36±6.85^●^

N=number, MAP=mean arterial pressure, SBP=systolic blood pressure, DBP=diastolic blood pressure, HR=heart rate.

^●^ *P*<0.05 compared with T0.

^★^*P*<0.05 compared with CC genotype.

### Effect of MDR1 gene polymorphisms on VAS assessment

Compared to VAS at 1 hour post-operation, all the mutations of the genotype of children with post-operative VAS showed a downward trend. There was no significant difference revealed between MDR1 3435C > T CC and CT + TT genotypes in terms of VAS at each time point (all *P* >0.05). Additionally, there was no significant difference observed among the different genotypes on MDR1 2677G > T/A (all *P* >0.05). Compared to the MDR1 1236C > T CC genotype, a significantly higher VAS was indicated in children who had the CT + TT genotype at 1, 2, 4, and 8 hours post-operation (all *P* <0.05), as demonstrated in Table 4.

**Table 4:** Comparison of VAS scores between different MDR1 genotypes patients$\left(\bar{x}\pm s\right)$

Geneotye	N	1h	2h	4h	8h
2677G>T/A					
TT	28	4.85±1.21	3.75±1.42^∆^	2.21±0.78^∆^	2.04±0.45^∆^
TG	108	4.77±0.98	3.42±1.05^∆^	2.54±0.92^∆^	2.08±0.43^∆^
GG	39	4.57±1.52	3.73±1.05^∆^	2.34±0.94^∆^	2.14±0.51^∆^
GA	34	4.73±1.25	3.32±1.21^∆^	3.02±0.74^∆^	2.22±0.61^∆^
TA	27	4.76±1.32	3.85±1.42^∆^	2.54±0.73^∆^	2.15±0.71^∆^
AA	4	4.61±1.35	2.84±1.02^∆^	2.84±0.56^∆^	2.56±0.64^∆^
1236C>T					
CC	30	3.94±0.97	3.21±0.65^∆^	2.01±0.42^∆^	1.69±0.42^∆^
CT+ TT	210	4.67±1.05^▲^	3.98±0.61^▲^^∆^	2.65±0.47^▲^^∆^	2.34±0.74^▲^^∆^
3435C>T					
CC	88	4.56±1.35	3.75±1.24^∆^	2.54±1.20^∆^	1.98±0.84^∆^
CT+ TT	152	4.61±1.24	3.84±1.36^∆^	2.34±1.04^∆^	2.02±0.87^∆^

注: N=number, VAS=Visual Analogue Scale.

^∆^*P*<0.05 compared with1h.

^▲^*P*<0.05 compared with CC genotype.

### Effect of MDR1 gene polymorphisms on Ramsay sedation score and FLACC assessment

As shown in Table 6, there was no significant difference between MDR1 3435C > T CC and CT + TT genotypes regarding the Ramsay sedation scores and FLACC scores after extubation for 5, 10, and 30 minutes (all *P* >0.05). No significant differences were observed among different genotypes on MDR1 2677G > T/A (all *P* >0.05). Compared to the MDR1 1236C > T CT + TT genotype, Ramsay scores at the 3 time points were reduced in children with the CC genotype, while FLACC scores were found to be higher (all *P* <0.05), as demonstrated in Table 5.

**Table 5:** Comparison of Ramsay sedation score and FLACC score between different MDR1 genotypes patients$\left(\bar{x}\pm s\right)$

Genotype	N	Ramsay	FLACC				
		5min	10min	30min	5min	10min	30min
2677 G>T/A							
TT	28	0.98±0.31	1.55±0.61	1.84±0.50	4.15±0.71	4.11±1.20	3.01±0.75
TG	108	0.99±0.32	1.62±0.57	1.75±0.52	4.29±0.73	3.74±1.04	3.19±0.58
GG	39	0.96±0.34	1.78±0.52	1.69±0.54	4.33±0.64	3.59±1.21	3.12±0.58
GA	34	0.98±0.35	1.79±0.65	1.66±0.54	4.26±0.68	3.54±1.17	3.24±0.45
TA	27	1.01±0.54	1.62±0.54	1.65±0.47	4.43±0.72	3.75±1.09	3.25±0.56
AA	4	0.78±0.52	2.04±0.86	1.26±0.51	4.76±1.01	4.05±0.89	3.54±0.61
1236C>T							
CC	30	0.57±0.54	1.13±0.34	1.24±0.41	4.48±1.06	4.49±1.15	4.15±0.86
CT+ TT	210	1.13±0.41^■^	1.55±0.51^■^	1.65±0.57^■^	3.91±0.94^■^	3.85±0.91^■^	3.15±0.65^■^
3435C>T							
CC	88	1.04±0.35	1.59±0.50	1.75±0.58	3.67±1.21	3.68±1.20	3.21±0.61
CT+ TT	152	1.03±0.36	1.57±0.54	1.73±0.61	3.63±1.15	3.64±1.09	3.26±0.65

^■^*P*<0.05 compared with CC genotype.

**Table 6:** Amplified primers of MDR mutations sites

Primer	5’→3’
2677 G>T/A	F:5’-TGCAGGCTATAGGTTCCAGG-3’
	R:5’-TTTAGTTTGACTCACCTTCCCG-3’
1236 C>T	F:5’-TCTTTGTCACTTTATCCAGC-3’
	R:5’-TCTCACCATCCCCTCTGT-3’
3435 C>T	F:5’-GCTGCTTGATGGCAAAGAAA-3’
	R:5’-ATTAGGCAGTGACTCGATGATGA-3’

F=forward, R=reverse.

### Adverse reaction

There were 8 cases of adverse reactions to anesthesia that occurred in this study. These included 2 cases of nausea and vomiting found in patients with the 2677 G>T/A CC genotype, 3 cases in patients with the 1236 C>TCT + TT genotype, and 2 cases in patients with the 3435 C>TCC genotype; 1 case of agitation in the recovery period was found with the 1236 C>T CC genotype. The comparisons of adverse reaction rate among different genotypes on MDR1 mutations were found to be nonsignificant (all *P* >0.05).

## DISCUSSION

Tonsil hypertrophy is a common pediatric disease. It may cause airway obstruction, which can affect sleep quality in children and promote the accumulation of CO2 in the lungs, possibly resulting in mental development abnormalities. Tonsillectomy is a common surgical procedure for tonsil hypertrophy [14, 15]. Tonsillectomy is performed inside the oral cavity; strict anesthesia requirements are needed since it can easily lead to throat irritation, and children often have a low degree of coordination during the surgery. To prevent adverse events during surgery, safe and effective anesthesia is of great significance not only for the operation but also for post-operative recovery [16, 17].

Recently, it was showndrug anesthetic or analgesic effect varies because of gene polymorphism [18, 19]. MDR1 is an important transporter that can constrain the accumulation of chemotherapeutic drugs [20]. The role of the 1236C > T, 2677G > T/A, and 3435C > T MDR1 gene polymorphisms on the anesthetic effects of the drug propofol combined with remifentanil was investigated in our study. The results we obtained revealed that the 1236C > T SNP contributed to individual variation in anesthetic effects following pediatric tonsillectomy. We determined that the patients with the MDR1 1236C > T CC genotype displayed superior anesthetic effects compared to patients with the CT + TT genotype. Takashina et al [21] study showed that MDR1 gene 1236C> T polymorphism can change remifentanil analgesic effect during the treatment of cancer pain, and found that patients of CC genotype have better results, which is consistent with our study. Analysis of the reasons may be due to patients of TT genotype need the largest dose of remifentanil and propofol, for they may accumulated in liver cells due to the decreased transport function of P-gp in patients of TT genotype, remifentanil and propofol are metabolized in the liver, resulting in increased dose of the drug needs.

Our study demonstrated that children with the CC genotype exhibited shorter times of induction, respiration recovery, eye-opening, and extubation compared to the MDR1 1236C > T CT + TT genotype. MDR1 is something that can be found in a variety of tissues including gastrointestinal and nasal respiratory mucosa, liver, kidneys, placenta, and the adrenal cortex [22]. It is known that Propofol and remifentanil have been extensively applied for anesthesia, analgesia, and procedural sedation [23-25]. The hemodynamic stability during anesthesia is often measured using MAP, SBP, DBP, and HR [26]. VAS, Ramsay, and FLACC scores are used to assess the pain scales after the emergence from anesthesia [27, 28]. Propofol-remifentanil anesthesia has a remarkable effect during tonsillectomy for its stable hemodynamics, short post-operative recovery time and high safety [5]. The study has also revealed that children with the CC genotype displayed significantly reduced MAP, SBP, DBP, and HR at T5 compared to the MDR1 1236C > T CT + TT genotype. Significantly higher VAS were indicated in children with the CT + TT genotype at post-operative 1, 2, 4, and 8  hours compared to the MDR1 1236C > T CC genotype. Ramsay scores were reduced in children after extubation for 5, 10, and 30 minutes with the CC genotype compared to the MDR1 1236C > T CT + TT genotype, while FLACC scores were found to be higher with the CC genotype.

We sought the cause and found that a critical impact on the therapeutic efficacy and the pharmacokinetics of drugs are varied in alterations in the expression and the activity of MDR1-encoded P-gp. Because of this, P-gp levels can influence the entrance of drugs into the cells. [29] Evidence suggests that elevated levels and enhanced activity of P-gp are conferred more by the CC genotype, while individuals with the TT genotype seem to have decreased P-gp activity. [30] Studies have found that genetic causes of MDR1 for the anesthetic effects are varied. MDR1 genetic variants rs12720464 and rs1055302 account for the individual variation of time of action in patients undergoing anesthesia with a single dose of rocuronium. Another study indicates that children who possess GG and GA genotypes of MDR1 rs9282564 present higher risks of opioid-related respiratory depression that lead to prolonged hospitalization. The minor allele (G) elevates the odds of a prolonged stay due to post-operative respiratory depression [31]. The experiment by Sia et al connects MDR1 1236C > T, 2677G > T/A, and 3435C > T to chronic pain in women receiving spinal anesthesia with intrathecal morphine for elective cesarean section. They revealed no significant difference in the total consumption of morphine, side effects, and pain scores among different genotypes. Women with the 3435C > T allele demonstrated a higher risk of suffering persistent post-operative pain [32]. Farhat et al’s [33] experiment indicated that managing nausea and vomiting during intravenous administration of ondansetron are all related to the MDR1 2677G > T/A TT genotype, which is similar to our findings. Our study confirmed that there is no significant association between the SNPs 2677G > T/A and 3435C > T that change anesthesia induction, emergence from anesthesia, hemodynamic changes, pain assessment, or adverse reactions.

However, the statistical analysis may have some limitations due to the small sample size, and it is necessary to enlarge the sample size in further study, such as increasing the number of genotypes samples. In addition, due to the limited number of samples, this study did not distinguish children from different ethnic groups, which is also need to analysis in the next study.

In conclusion, it can be said that our study gives evidence that the MDR1 SNP 1236C > T is partly the reason for single differences in the anesthetic effects that follow pediatric tonsillectomy. Additionally, children who possess the MDR1 1236C > T CC genotype were shown to have superior anesthetic effects in terms of anesthetic induction and restoration, hemodynamic changes, pain and sedation assessment compared to children who possess the CT + TT genotype.

Since the time and budget were limited, it was impossible to conduct the experiments in a larger population. In addition, we believe that further analysis of interactions between varieties of gene polymorphisms in populations from different regions and of different races in China would be significant and warrants in further study.

## MATERIALS AND METHODS

### Subject and grouping

Two hundred and forty children, including 142 males and 98 females, received tonsillectomy with general propofol–remifentanil anesthesia at Zhejiang provincial people’s hospital from August 2012 to September 2016. All the children recruited for this study were between the ages of 3 and 12 years with a mean age of 7.0 5± 2.30 years, and they weighed between 13 and 45 kg. The mean time to complete the operation was 44.85±8.15 minutes. The involvement criteria for eligible patients were as follows: people who underwent post-operative analgesia voluntarily and people who had no allergy history to any kind of anesthetic drugs or no operation contraindication. The non-involvement criteria for non-eligible patients were as follows: heart block or cardiac abnormalities, liver or renal dysfunction, drug abuse history, and allergy to any kind of drug used in this study. This experiment was approved by the Ethics Committee of Zhejiang provincial people’s hospital. Informed consent for anesthesia was obtained from the parents or legal guardians.

### Methods

All children were anesthetized with intravenous 0.1 mg / kg dexamethasone and 0.01 mg / kg scopolamine. Target-controlled infusion pump induction of anesthesia was activated. Remifentanil (Yichang people Fook Pharmaceutical Co., Ltd., batch number: 20140197) and propofol (Sichuan Guorui Pharmaceutical Limited liability company, batch number: 20150824) initial doses were set at 2.5 μg / L and 2.5 mg / L, respectively, after imputing parameters such as the child’s weight, age and others. Intravenous infusion of 0.1 mg / kg vecuronium by endotracheal intubation then followed. The bispectral index was maintained at 45-55 during surgery. The propofol concentration was adjusted at any time to maintain the mean arterial pressure (MAP) at 60 ∼ 90 mmHg and the heart rate at 80 ∼ 120 beats / min. The remifentanil concentration was adjusted to maintain the stability of anesthesia, and vecuronium was used as appropriate. Remifentanil infusion was ceased at the end of the surgery and propofol infusion stopped 5 minutes after the surgery. The ventilator was cleared when children began to breathe, cough, and swallow normally. The patient was transferred to the observation room at 5 minutes after the oxygen supply stopped, and the percutaneous oxygen saturation (SPO2) stabilized at approximately 95%.

Hemodynamics after anesthesia: The times taken for the following activities were recorded: time of anesthesia induction, eye-opening, respiration recovery, and extubation. Mean arterial pressure (MAP), diastolic blood pressure (DBP), systolic blood pressure (SBP), and HR of different genotypes were recorded and compared at 5 minutes after repose (T0), 0 minutes after tracheal intubation (T1), 5 minutes after tracheal intubation (T2), 0 minutes after tonsillectomy (T3), 0 minutes after removal of mouth-gag (T4) and 5 minutes after extubation (T5).

Analgesia and adverse reaction observation: At 1, 2, 4, and 8 hours post-operation, the visual analog scale (VAS) was recorded [12]. The Ramsay sedation score was assessed at 5, 15, and 30 minutes after extubation: 1 point for anxiousness or restlessness; 2 points for cooperativeness and tranquility; 3 points for responding to commands with unclear murmurs; 4 points for drowsiness and brisk response to calling; 5 points for drowsiness and sluggish response to calling; and 6 points for deep sleep or anesthesia [13]. The Face, Legs, Activity, Cry, and Consolability (FLACC) pain assessment was assessed at 5, 15, and 30 minutes after extubation: 0 points for content and relaxed; 1 to 3 for slight pain; 4 to 6 for medium pain; and 7 to 10 for severe pain and uncomfortable. We also observed the adverse reaction rate post-operation followed by consciousness.

Peripheral blood sampling and DNA extraction: Fasting venous blood (3 mL) was sampled and placed into a sodium citrate anticoagulation tube labeled with the subject number and name. Using a DNA extraction kit (Article No., 52304; Qiagen Company, Hilden, Germany), the blood was centrifuged at 3000 rpm/min for 10 minutes to extract whole blood DNA from the peripheral blood. Fasting venous blood (3 mL) was sampled and placed into the sodium citrate anticoagulation tube labeled with the subject number and name. Using a DNA extraction kit (Article No., 52304; Qiagen Company, Hilden, Germany), the blood was centrifuged at 3000 rpm/min for 10 minutes to extract whole blood DNA from the peripheral blood and stored at −80°. We detected 2677 G>T/A, 1236 C>T and 3435 C>T mutations in the MDR1 gene based on extracted genomic DNA. The sequencing primers are shown in Table 6. The sequencing results were opened and analyzed using Chromas (version 2.41) software.

### Statistical analysis

Statistical analysis was performed using SPSS 19.0 (SPSS Inc., Chicago, IL). Measurement data were expressed as the mean ± standard deviation (± s), and comparisons between two groups using were tested using the t test. Counting data was expressed as [n (%)], and the χ2 test was used for statistical analysis with a significance level of 0.05. P <0.05 was statistically significant.
